# The role of veterinarians in equestrian sport: A comparative review of ethical issues surrounding human and equine sports medicine^☆^

**DOI:** 10.1016/j.tvjl.2013.05.021

**Published:** 2013-09

**Authors:** Madeleine L.H. Campbell

**Affiliations:** Department of Production and Population Health, The Royal Veterinary College, Hawkshead Lane, North Mymms, Hertfordshire AL9 7TA, UK

**Keywords:** Veterinary ethics, Equine sports medicine, Conflicts of interest, Evidence-based veterinary medicine, Equine welfare

## Abstract

Veterinarians have a key role in providing medical care for sports horses during and between competitions, but the standard client:veterinarian relationship that exists in companion and production animal medicine is distorted by the involvement of third parties in sports medicine, resulting in distinct ethical dilemmas which warrant focused academic attention. By comparing the existing literature on human sports medicine, this article reviews the ethical dilemmas which face veterinarians treating equine athletes, and the role of regulators in contributing to or resolving those dilemmas.

Major ethical dilemmas occur both between and during competitions. These include conflicts of responsibility, conflicts between the need for client confidentiality and the need to share information in order to maximise animal welfare, and the need for an evidence base for treatment. Although many of the ethical problems faced in human and equine sports medicine are similar, the duty conferred upon a veterinarian by the licensing authority to ensure the welfare of animals committed to his or her care requires different obligations to those of a human sports medicine doctor. Suggested improvements to current practice which would help to address ethical dilemmas in equine sports medicine include an enhanced system for recording equine injuries, the use of professional Codes of Conduct and Codes of Ethics to establish acceptable responses to common ethical problems, and insistence that treatment of equine athletes is evidence-based (so far as possible) rather than economics-driven.

## Introduction

Although public concern about the use of horses in sport is not new (Higgins, 1996), it has increased in recent years. Heightened public and academic awareness of the welfare issues surrounding equestrian events including (but not limited to) racing, eventing, endurance, dressage, show-jumping, reining and polo (McLean and McGreevy, 2010) was reflected in the media outcry about deaths and injuries of horses in the 2011 and 2012 British Grand National steeplechase.^1^

Veterinarians have a key role in providing medical care for horses at all times. Like human sports medicine doctors, veterinarians treating elite equine athletes face a potential conflict between their duty to safeguard the welfare of the athlete under their care, and their responsibility to the trainer/manager/owner of that athlete (and, in the human case, the athlete himself) who are purchasing medical care and have an interest in keeping the athlete competing. Increasing recognition amongst the veterinary profession of the particular ethical issues associated with equine sports medicine was reflected in the inclusion of a session entitled ‘*Ethics, Scope of Practice and Racing*’ at the 2012 convention of the American Association of Equine Practitioners.^2^

This article reviews the ethical dilemmas which face veterinarians treating equine athletes, and the role of regulation in contributing to or resolving those dilemmas. The focus is not on the moral question of whether horses should be used for sport at all (see, for example, Campbell, 2013), but rather on the ethical issues facing veterinarians when the use of horses for sport is permitted by society and by law. Significant ethical dilemmas face veterinarians between as well as during competitions. If veterinarians are to safeguard not only the welfare of the animals under their care but also the integrity of the veterinary profession, they must play a proactive role in (1) identifying and addressing welfare issues, (2) researching methods of reducing sports-related injuries, and (3) ensuring that the treatment of equine athletes is evidence-based rather than economics-driven.

## Ethical issues surrounding the use of horses for sport, and the role of the veterinarian

### Distortion of the standard veterinarian:client relationship

Although a substantial body of work on welfare issues surrounding the use of horses in sport exists, and examples are to be found in both the interested lay press^3^,^4^ and the scientific literature (Jeffcott et al., 1982; Lam et al., 2007; Ely et al., 2009; Campbell, 2012; Reed et al., 2012),^5^ relatively little academic or public attention has been paid to the ethical dilemmas faced by veterinarians working in equestrian sport, or to the role of veterinarians in addressing some of the issues.

All practising veterinarians carry responsibilities not only to animals but to owners, society and practice partners (Rollin, 1978; Main, 2006, 2011; Yeates, 2009). The standard relationship between veterinarian and client often does not apply with competition horses, when the veterinarian may be obliged to relate not only to the owner, but also to a trainer, a rider, a team manager, and selectors. This exacerbates the potential conflicts of interest faced by all veterinarians, and justifies focused academic consideration, as research into as the ethics of sports medicine does within human medical ethics (Murthy et al., 2012).

### Ethical dilemmas facing veterinarians during competitions

The ethical issues confronting veterinarians include problems of confidentiality, owner/trainer’s unrealistic expectations of treatment, the use and abuse of medication, and conflicts of duty to the horse and its human connections. These are broadly analogous to those faced by doctors working in human sports medicine (Anderson and Gerrard, 2005). However, unlike human sports medicine, there has been little analysis in the veterinary literature of how these issues might be effectively addressed. This applies particularly to the dilemmas encountered by and the behaviour of veterinarians before, after or between (rather than during) competitions.

During a competition, at least at elite level, the duties and obligations of veterinarians are well-defined by regulatory bodies. Thus, for example, the Fédération Equestre Internationale (FEI) publishes Veterinary Regulations which are updated annually.^6^ These rules define the categories of veterinarians at FEI events, and detail their responsibilities. Similarly, the British Horseracing Authority’s (BHA) Equine Science and Welfare Department sets the requirements for experience and competence of racecourse veterinarians, and BHA veterinary officers are present on race days to advise Stewards, monitor equipment and work with treating veterinarians who are providing clinical care.

Regulations in racing and other equine sports extend to the administration of therapeutic drugs and to drug-testing (Toutain, 2010), so that the responsibilities of veterinarians are clearly defined, and decisions about what constitutes ethical behaviour are consequently relatively simple to make. However, these responsibilities may be less well defined at low-level competitions. Mechanisms by which recommendations for ethical good practice for event veterinarians can be transferred from elite to grass-roots level warrant further research.

### Ethical dilemmas facing veterinarians between competitions

Unlike the intra-competition period, regulations and advice from sports governing and professional bodies about how veterinarians ought to deal with ethical dilemmas which occur outside of competition is limited. The exception to this generalisation is advice about medication between competitions, which does exist. The FEI maintains a searchable on-line database of prohibited substances,^7^ and both the FEI and BHA publish lists of drug detection times. Interestingly, there is a divergence of ethical approach towards medication adopted by the BHA and the FEI. The BHA stance is that it is never in the best interest of a horse to be raced whilst under the effect of medication,^8^ whereas the FEI believes that there is some welfare benefit in allowing horses to compete on specified, ‘permitted’ medications, e.g. anti-ulcer drugs including omeprazole, most antibiotics, topical wound ointments (not including corticosteroids) and ‘*preventative or restorative joint therapies*’ administered by some routes.

Veterinarians are bound by law and by guides to professional conduct to act within relevant legislation and local rules. The potential for disciplinary and legal proceedings to result from failure to act within rules was illustrated by the disciplinary case of the UK’s Royal College of Veterinary Surgeons (RCVS) vs. Main (2011), in which one of the charges against the defendant was that he had injected a horse with a substance on the day of a race when he either knew or ought to have known that to do so contravened BHA rules.^9^

Other than medication regulations, however, there is little advice available to veterinarians on ethical decision making surrounding between-competition treatment of equine athletes. The goal of treating athletes (short term performance) differs from the usual goals of treating companion animals (quality of life across years, longevity and freedom from disease and/or pain). It also differs from the usual aims of maximising productivity and minimise welfare insults for production animals. There is an overriding, economically-driven requirement in both equine and human sports medicine to return the athlete to competition as soon as possible. Combined with the complexity of responsibilities to the animal–owner–trainer–team axis, this causes ethical pressures on veterinarians that are unique to sports medicine and encompass issues of autonomy, confidentiality, and rationale for treatment.

### Conflicts of responsibility

In human medicine, the concept of autonomy refers to a person’s right (providing that he is adequately informed, capable of understanding, and rational) to make decisions about what happens to him, and to have those decisions respected by others (Beauchamp and Childress, 2009). Autonomy is a problem in human sports medicine (Dunn et al., 2007) when the patient’s right to make informed decisions about his own treatment free of the influence of others may be compromised by the interests of team owners and managers who wish the player to keep competing.

Discussions about whether patient autonomy can exist when the patient is an animal (see, for example, Chan and Harris, 2011) are outside the scope of this paper. Nevertheless, autonomy may be a problem in veterinary sports medicine when the owner’s, trainer’s and indeed the veterinarian’s rights to make an autonomous decision conflict. The trainer has an interest in keeping the horse in training and returning it to competition as soon as possible. The owner may or may not share that interest. Veterinarians in UK admitted to the RCVS promise that: ‘*above all (their) constant endeavour will be to ensure the health and welfare of animals committed to (their) care*’.^10^ The veterinarian therefore ought to treat the horse to ensure maximise welfare, which might involve a period of rest that is unacceptable to a trainer.

In a survey of human sports medicine doctors (Anderson and Gerrard, 2005) the conflict between doctors’ autonomous right to treat the athlete with the aim of maximising recovery (welfare) and the autonomous right of managers/trainers to decide on the most appropriate treatment for players in their employment was identified by half of the respondents as a major ethical dilemma. A similar dilemma exists for veterinarians whose right to make an autonomous decision about best treatment aimed at maximising long-term welfare conflicts with the owner or trainer’s right to make autonomous decisions about their animals. The veterinarian may have different priorities from the owner or trainer, and an assistant and partner veterinarian might have different priorities from each other according to considerations of practice viability and financial responsibility to other members of the practice, particularly if it is dependent upon work as a team or a trainer’s regular veterinarian (Anderson and Gerrard, 2005).

Dunn et al. (2007) argued that in human sports medicine a physician’s reputation may be built on ability to effect short-term repair or to improve performance, rather than on long-term preservation of the athlete’s health, and that this might influence a clinician’s decision making process to the detriment of the patient’s long-term welfare. Media exposure may also exert pressure, and the kudos surrounding treating a high-profile patient might persuade clinicians to treat beyond their expertise (Murthy et al., 2012). Similar pressures are likely to exist in equine medicine, where owners/trainers may expect the veterinarian to treat the horse to optimise performance. Rumours and anecdote abound regarding the administration to horses by veterinarians of intra-articular medications or other treatments such as intravenous infusions, which are not currently detectable on routine dope tests. Unsurprisingly, most such rumours are unsubstantiated.^11^ There may anyway be difficulty in differentiating between excessive treatment and legitimate, routine maintenance of elite athletes. This distinction may be wilfully blurred when efforts are being made to turn mediocre animals into more successful performers. Variation in medication rules between international racing jurisdictions further confuse the situation (Higgins, 1996) and provide the façade of an (unacceptable) excuse for such behaviour.^12^ The relationship between such pressures and clinical decision-making processes, including whether to refer and the influence of insurance^13^ on treatment decisions, requires further investigation.

The conflict between the veterinarian’s responsibility to the horse and to the trainer/team who employ the veterinarian is analogous to a doctor’s conflicting loyalties to the athlete and to the team with whom the doctor has a contract (Anderson and Gerrard, 2005). Even in human medicine, in extreme examples, such conflict can result in medical harm being caused to the patient, as in the case of a doctor who (at the player’s request) deliberately injured a player in order that a substitution might be made by his team.^14^

However, there is a significant difference between human and equine sports medicine because horses, unlike human athletes, are unable to express their views. Consequently, given his duty to prioritise the welfare of the animal under his care, where the veterinarian’s assessment of what is best for the health of the athlete differs from the preferred solution of the owner/trainer the veterinarian *ought* to act as the animal’s advocate.

Interestingly, in one survey (Anderson and Gerrard, 2005), 28% of medics listed *themselves* as one of those to whom they were responsible when treating athletes. This conflates the proposal that conflicts between autonomy of owners, trainers and veterinarians can be stressful, much as ethical dilemmas cause stress for veterinarians in general practice (Batchelor and McKeegan, 2012). Dunn et al. (2007) asked how a doctor can recognise that the team has a legitimate interest in outcome and yet remain loyal to the patient? For a veterinarian, the equivalent question is how he can recognise that the owner/trainer/team has a legitimate interest in the outcome of treatment, and yet fulfil his obligation to safeguard the welfare of animals under his care.

Codes of Professional Conduct such as those of the RCVS and the American Veterinary Medical Association (AVMA) fulfil a useful role here in establishing systems of addressing common ethical dilemmas which can both ‘*protect practitioners from unacceptable demands and external pressures*’ (Anderson, 2009) and be used as a yardstick against which actions can be measured, for example during a disciplinary hearing.

### Patient confidentiality and information sharing

Issues about autonomy and conflicts of loyalty in sports medicine carry associated questions of patient confidentiality (Murthy et al., 2012). When there are many layers to the client–veterinarian relationship decision-making may be delayed, and the veterinarian may be unsure about who is and is not entitled to share in patient information which ought normally to remain confidential.^15^ This ethical dilemma was recognised in the development of the AAEP’s protocol to improve transparency and communication in the owner–trainer–veterinarian relationship.^16^

Where many people are acting as owners or owner’s agents, and where multiple veterinarians become involved in an animal’s care, problems develop not only of client confidentiality, but also, conversely, through compromising animal welfare by failing to share medical information This can occur during routine treatment if one trainer employs multiple veterinarians, particularly if those veterinarians are competing with one another for the work. Although the RCVS (and other) Codes of Professional Conduct encourage transfer of clinical information between veterinarians, client confidentiality must also be protected, and it is in any case difficult for veterinarians to exchange information if the trainer/owner does not make them aware that there are several veterinarians caring for one animal.

Under current rules governing elite equestrian sport, veterinarians have a duty to ensure that the owner/trainer/rider of the horse is fully informed about the implications for the rules of competition concerning any therapeutic drugs, and to record that information accurately, but they are not expected to inform regulators directly if such treatment has been given. Under BHA rules, the trainer remains strictly liable if a horse fails a drugs test. However, the 2010 ‘Clean Sport’ regulations of the FEI for the first time designated veterinarians as potential ‘additional responsible persons’ (the rider being the main ‘Person responsible’ and as such strictly liable), thus raising the possibility that veterinarians could have their FEI accreditation removed and be penalised under certain circumstances if found to have contributed to a horse testing positive.

The maintenance of a Medication Logbook required by FEI medication control regulations (2010) goes some way to creating a system which records in one place details of those medications that have been given to a horse, and provides access by interested parties. However, the use is limited by the fact that, although prohibited substance administration *should* be recorded, the recording of non-prohibited medications is voluntary.

The potential responsibility of veterinarians to share information about injuries (rather than drugs) is ambiguous. Although the RCVS requires that veterinarians keep clinical records, there is no requirement by sports governing bodies to record injuries which occur outside of competition or any (non-medicinal) treatment. Although the BHA collects and analyses information about injuries and fatalities during racing, and the FEI is developing a programme of surveillance of injuries which occur during competition, systems enabling veterinarians to record injuries which occur *between* competitions appear to be lacking.^17^ The transmission of information about injuries between private and team veterinarians may be hampered if a rider, rather like a human athlete (Anderson and Gerrard, 2005) is aware of an injury but chooses not to divulge that to the team veterinarian for fear of jeopardising a team place.

In human medicine in the USA, a doctor has a duty to reveal confidential information (e.g. a cardiac condition in a race driver) when failure to do so may expose others to harm (Murthy, 2012). Risks of harm might be associated with some non-medicinal equine treatments, for example if the insensitivity caused by a neurectomy performed to mask lameness causes a horse to stumble and throw the rider, or injure spectators. Competing with an injured horse could also of course compromise the welfare of that horse. In the absence of a regulatory mechanism dictating that private veterinarians must record injuries and non-medicinal treatments in a manner accessible to regulators or team veterinarians, the need to protect client confidentiality probably overrides any sense of responsibility to those individuals. This does not excuse, however, the veterinarian’s responsibility for the welfare of the horse, for example in UK under the RCVS Professional Codes of Conduct and the Animal Welfare Act (2006).^18^ Thus the veterinarian who knows that an animal is injured or not fully recovered and that the owner/rider nonetheless intends to compete it has a potential conflict of interest between his duty to the animal’s welfare and to client confidentiality.

The animal’s welfare should always be paramount, but this is complicated when the veterinarian’s income is dependent upon retaining clients. Although the FEI Clean Sport regulations (2010) suggest that veterinarians should contribute to decisions about whether a horse is unfit to compete, in the absence of any notification mechanism it remains possible for owners/trainers/riders to obscure and indeed to ignore such veterinary advice. Improved regulation covering the recording of injuries and/or non-medicinal treatments and the obligatory transmission of that information to regulatory authorities would improve the welfare of competing horses, and protect veterinarians from potential conflicts of interest and concerns about client confidentiality.

### Ethics and evidence-based medicine

Questions about how veterinarians should act ethically between competitions relate closely to the issue of evidence-based veterinary medicine (Anon, 2012). In the UK, the Animal Welfare Act (2006) prohibits causing unnecessary suffering. Combined with the veterinarian’s duty to safeguard the welfare of any animal under his care, this ought to ensure that no procedure is undertaken which causes (even temporary) harm, unless the harm is necessary in the sense that a benefit is reasonably expected to result from it.

The need to avoid causing unnecessary harm (non-maleficence) is an accepted tenet of human medicine which ought to apply to veterinary medicine, and which is frequently underwritten by an implicit cost:benefit analysis of proposed treatments. For example, each time a veterinarian vaccinates a horse he is causing harm, since the injection is painful and handling may be stressful, but that is outweighed by the perceived benefit of protection against disease. However, the debate about (for example) thermocautery or ‘firing’ of equine tendons as a treatment for tendon injury provides a current example of refusal by some veterinarians to adopt such a cost:benefit approach in clinical practice. In 1983, a study of the ‘pathology of tendon injury and repair, especially after firing’ was published (Silver et al., 1983). The authors concluded that ‘*On the basis of the pathological and biochemical evidence…. line ‘firing’ cannot be considered a desirable or effective treatment of acute or chronic equine tendon injury*’. This study has recently been cited as constituting ‘*the final verdict of non-effectiveness (and detriment to welfare)… on the medieval technique of tendon firing*’ (van Weeren, 2012).^19^

Nonetheless, despite the lack of evidence of efficacy for a ‘treatment’ which is painful and deforming, and the RCVS having recently adopted a robust stance against firing,^20^ some members of the veterinary profession remain prepared to undertake and defend the procedure^21^ (Harris, 2012; Jepson, 2012).

A lack of evidence base for treatments is not uncommon in sports medicine, even in the human field where technology advances rapidly and evidence-based medicine lags behind (Dunn et al., 2007). Where the overriding objective is to return the athlete (human or equine) to athletic function as soon as possible, there are inevitable economic pressures to use unproven treatments in the hope of a ‘quick fix’. Additional pressure to use a particular unproven treatment may be brought to bear on the veterinarian by owners/trainers who believe that it confers a competitive advantage.

Dunn et al. (2007) proposed that the ethical duty of a medical doctor when a treatment is unproven extends only to ensuring that the athlete is fully informed, and then leaving the athlete to exercise his autonomous choice. Because they are unable to express autonomous choices, this is insufficient in the case of equine athletes, especially since the trainer/owner may make decisions which are not in the horse’s best welfare interests. Thus the veterinarian, unlike the doctor, has a responsibility when faced with a lack of evidence to act as an advocate for the animal. This does not necessarily preclude recommending a treatment the efficacy of which is unproven due to its novelty and small patient numbers. However, it does surely preclude offering or agreeing to a request from a trainer to undertake a treatment such as firing which is painful, causes a visible harm, and which has not been proven to offer any therapeutic benefit exceeding that of alternative, less painful treatments. In such a situation it cannot be reasonably assumed that the harm/pain will be outweighed by the benefit, and thus any suffering caused would be unnecessary, and, in the UK for example, would contravene the Animal Welfare Act (2006).

Evidence based veterinary medicine – particularly equine medicine, where numbers are small – may not always be possible. Although one might speculate that the pressures to treat rapidly in equine sports medicine result in quicker translation of research into practice than in other branches of veterinary medicine, this is unproven. The translation of research into practice is problematic in human medicine (Zerhouni, 2009) and likely to be further limited in veterinary medicine (Toews, 2011). Notwithstanding such limitations, it is unethical to undertake harmful procedures in defiance of what evidence *is* available, or solely because the owner/trainer requests it. It is nonetheless interesting to note that divisions within the veterinary profession in the UK over the issue of firing reflect the description by Anderson (2009) of inconsistent attitudes between human sports doctors about when it is and is not ethical to undertake client-requested treatments or actions. There is a role here for professional Codes, which can provide authority to a veterinarian who is refusing to undertake a treatment despite the owner/trainer’s insistence that he do so, and also for voluntary ‘Codes of Ethics’ (Anderson, 2009) to be agreed by those working in particular circumstances, for example as racecourse veterinarians.

Anecdotally, the public example of one member of the profession having been disciplined for breaking professional Codes of Conduct (for example the much-publicised removal of a veterinary surgeon from the RCVS register for having back-dated equine vaccination certificates^22^) can make it subsequently easier for other members of the same profession to resist pressure from owners/trainers to act in the same way.

Evidence-based science depends upon research, and investigative work by veterinarians into methods of preventing, reducing and treating sporting injuries in equine athletes (see, for example, Weller et al., 2006; Nagy et al., 2010; Clegg, 2012; Kalisiak, 2012; Nagy et al., 2012; Reed and Leahy, 2013) is an important aspect of the profession’s moral responsibility to animals under their care, and of demonstrating concern for animal welfare (Peter, 2010). Mechanisms by which research, particularly if carried out under proper regulated legislation, are translated into ‘accepted practice’, and the ethics of practice-based clinical research are areas both requiring further study.

## Conclusions

The position of veterinarians treating sports animals differs from that of veterinarians in companion or production animal practice, and deserves particular ethical consideration. Codes of Professional Conduct and Codes of Ethics have a useful role in establishing acceptable responses to common ethical dilemmas, and by so doing protecting veterinarians against pressure from those who may not be acting in the horse’s best interests, and against accusations of unethical conduct. The continued development of such Codes by bodies to which veterinarians working in sports medicine are affiliated would be beneficial.

Systems for recording and sharing information about drug administration are better developed than for injuries. To fulfil their stated aim of protecting the welfare of competition horses, governing bodies should develop compulsory systems for recording injuries (both during and between competitions) designed to enable veterinarians to provide relevant information without compromising client confidentiality. It is incumbent upon governing bodies of all equine disciplines to transparently collect, collate and analyse such data, and to make it publicly available. Such information is crucial in establishing an evidence base about the incidence, types and causes of injuries in competition horses both during and between competitions. The role of veterinarians in using such data to undertake research which prevents, reduces and treats equine sporting injuries is an ethically important one, and further study is required into the most effective ways of translating such research into practice.

## Conflict of interest statement

The author of this paper does not have a financial or personal relationship with other people or organisations that could inappropriately influence or bias the content of the paper.

## Footnotes

1 Another National Tragedy, The Daily Mail on-line, http://www.dailymail.co.uk/news/article-2129727/Grand-National-2012-horse-deaths-notorious-Aintree-jump-included-Gold-Cup-winner.html, accessed 10 January 2013; BBC attacked for ‘covering up’ Grand National deaths, The Telegraph on-line, http://www.telegraph.co.uk/news/uknews/8440520/BBC-attacked-for-covering-up-Grand-National-deaths.html, accessed 10 January 2013; Aintree Day of Horror, The Daily Mail on-line, http://www.dailymail.co.uk/sport/racing/article-1375310/Grand-National-2011-Aintree-day-horror-millions-2-horses-die.html, accessed 10 January 2013.

2 Ethics, Scope of Practice and Racing. Proceedings of the 2012 Convention of the AAEP, pp. 59–84. http://www.ivis.org/proceedings/AAEP/2012/toc.asp. Accessed 07 May 2012.

3 Maas, A., 2012. Drug use – and abuse – in horses. Examiner.com. http://www.examiner.com/article/drug-use-and-abuse-horses. Accessed 10 January 2013.

4 World Horse Welfare, 2013. The whip in racing. http://www.worldhorsewelfare.org/help-tomorrow/the-whip-in-racing. Accessed 10 January 2013.

5 Rhoden, W.C., 2008. Wondering if steroids fuelled a run at glory. The New York Times. http://www.nytimes.com/2008/06/08/sports/othersports/08rhoden.html?ref=horseracing&_r=0. Accessed 10 January 2013.

6 See: http://www.fei.org/veterinary/veterinary-regulations. Accessed 10 January 2013.

7 See: http://www.feicleansport.org/prohibited.html. Accessed 27 July 2012.

8 Although traces of antimicrobials or anthelmintics may be present on race days. See: http://www.britishhorseracing.com/resources/equine-science-and-welfare/medication-and-doping-faqs.asp#10. Accessed 05 February 2013.

9 See: http://www.rcvs.org.uk/document-library/main-feb-2011-decision/. Accessed 07 May 2013.

10 See RCVS, 2012. http://www.rcvs.org.uk/advice-and-guidance/code-of-professional-conduct-for-veterinary-surgeons/#declaration. Accessed 04 July 2012.

11 Occasionally, however, the role of the veterinarian is subjected to scrutiny; see, for example, http://www.independent.co.uk/sport/general/coral-cove-findings-cast-doubt-on-the-vet-741672.html. Accessed 7 May 2013.

12 As in the 2013 case of anabolic steroid abuse in racehorses. See: http://www.horseandhound.co.uk/news/eleven-godolphin-horses-test-positive-for-banned-substances and http://www.bbc.co.uk/sport/0/horse-racing/22295639. Accessed 07 May 2013.

13 This may be a more significant issue at lower levels of competition since many elite horses are not insured.

14 See: *Doctor cut lip of Harlequins winger Tom Williams*: http://www.bbc.co.uk/news/uk-england-london-11055639. Accessed 23 January 2013.

15 RCVS, 2012. Code of Professional Conduct: client confidentiality (section 14.1). See: https://www.rcvs.org.uk/advice-and-guidance/code-of-professional-conduct-for-veterinary-surgeons/. Accessed 10 January 2013.

16 See: AAEP examines owner–trainer–vet relationship. The Bloodhorse Magazine on-line. http://www.bloodhorse.com/horse-racing/articles/66611/aaep-examines-owner-trainer-vet-relationship. Accessed 10 January 2013.

17 The BHA does have a system which allows trainers and veterinarians to contribute veterinary data from training to the ‘Training and animal welfare system database’. However, the effectiveness of this system in enabling veterinary surgeons to record information without being pressurised by clients not to do so or fearing compromising client confidentiality is likely to be limited by the fact that the system is voluntary.

18 http://www.legislation.gov.uk/ukpga/2006/45/crossheading/prevention-of-harm. Accessed 07 May 2013.

19 A study by O’Meara et al. (2010) was cited during a debate at BEVA Congress 2012 as providing evidence that ‘firing’ is an efficacious treatment. This is not the case. The study purported to assess the effect of firing, intra-lesional insulin-like growth factor-type 1, and superior check ligament desmotomy on re-injury, level of performance and number of races competed in post-injury. The results showed that treated horses were *less* likely than untreated horses to compete in five races following the return to training. Unfortunately, there was no control group of injured horses which were treated only with rest. Without such a control group, the results, which supposedly showed that treated horses performed as well as (uninjured) control horses for various parameters, do not in fact prove that ‘firing’ is an efficacious treatment, because not treating (except with rest) might have had the same results. Furthermore, the effect of the three separate treatments on the parameters measured was not separated out, making it impossible to assess the specific effect or efficacy of firing.

20 J. Parker, Letter to the *Veterinary Times*, 3 December 2012. Firing letter ‘omitted important facts’.

21 J. Molyneux, Letter to the *Veterinary Times* 17 December 2012. (The RCVS has been against firing since 1983).

22 Top Vet struck off over date discrepancy. Horse and Hound Magazine on-line. IPC Media. http://www.horseandhound.co.uk/news/397/109104.html. Accessed 20 January 2013.
